# Re-imagining crisis care: experiences of delivering and receiving the Assured brief psychological intervention for people presenting to Emergency Departments with self-harm

**DOI:** 10.3389/fpsyt.2024.1271674

**Published:** 2024-03-26

**Authors:** Neha Shah, Sally O’Keeffe, Sam Hayward, Mimi Suzuki, Rose McCabe

**Affiliations:** ^1^ School of Health and Psychological Science, City, University of London, London, United Kingdom; ^2^ Population Health Sciences Institute, Newcastle University, Newcastle-Upon-Tyne, United Kingdom

**Keywords:** Emergency Department, liaison psychiatry, self-harm, suicide, qualitative research, solution-focused, psychological intervention

## Abstract

**Background:**

Risk of suicide is increased immediately following emergency department (ED) attendance for self-harm. Evidence suggests that brief psychological interventions delivered in EDs are effective for self-harm. The Assured intervention comprises an enhanced biopsychosocial assessment in the ED, collaborative safety planning and three rapid solution focused follow-up sessions.

**Aim:**

We addressed the following research questions: What were ED mental health liaison practitioners’ and patients’ experiences of the Assured intervention? What were the barriers and facilitators? What might the mechanisms be for improving experiences and outcomes?

**Methods:**

We conducted a feasibility study of the Assured intervention in four EDs in Southeast England. Semi-structured interviews were conducted with 13 practitioners and 27 patients. Interviews were transcribed, coded line-by-line in Nvivo and thematically analysed using an inductive approach. Inter-rater reliability was calculated with a kappa coefficient of 0.744.

## Highlights

Five overarching themes were identified:

- The intervention mostly gives agency and hope to highly distressed patients, and helps patients implement strategies to support their mental health; however potential adverse effects were patients becoming overwhelmed or triggered.
- The intervention works by creating a space for patients to express what is important to them; facilitating insight and reflection, validating emotions, and building self-esteem, helping patients manage boundaries and trial what works to support their mental health.
- The intervention re-imagines the practitioner-patient relationship and facilitates trust and new possibilities for patients who have difficult relationship and help-seeking histories.
- The intervention requires a significant shift in professional culture and a new way of working practically by doing follow-up work, moving from a reactive to a more proactive role. This needs support and supervision but is rewarding for practitioners.
- The intervention challenges the limitations of point-of-crisis care by opening up a new and timely therapeutic space and holding and supporting patients to access further support.

Providing rapid therapeutic follow-up support after attending ED with self-harm/suicidality can begin to address existing failures in the mental health system, where patients feel unsupported and - at times - judged by professionals, by supporting people and fostering agency. However, there are significant cultural and service pathway barriers.

## 1 Introduction

Suicide is a leading cause of death (1) with 800,000 people dying by suicide each year globally (2). The strongest risk factor for suicide is self-harm, which refers to intentional self-poisoning or self-injury, irrespective of motive or the extent of suicidal intent (3). One in 25 people who present at hospital for self-harm die by suicide within the subsequent 5-years (4), with the risk of suicide greatest in the initial week after discharge from hospital (5). Strict referral criteria for mental health services means that patients who self-harm are often unable to seek help elsewhere (6, 7). This makes the ED a lifeline for patients who self-harm, who are known to be at increased risk of suicide. There are an estimated 220,000 self-harm presentations each year in EDs in England (8). International evidence has found brief psychological interventions in EDs to be effective in reducing self-harm and suicide (9). The UK Government’s Suicide Prevention Strategy identified those presenting to EDs with self-harm as a priority group (10), yet the evidence base for interventions in the NHS context in the UK is sparse.

EDs in England have a psychiatric liaison team staffed by specialist mental health practitioners. NICE guidelines state that patients presenting to EDs with a mental health issue should be referred to the psychiatric liaison team for a comprehensive psychosocial assessment with a mental health practitioner (3). This includes comprehensive assessment of the patient’s needs and risk of harm, and aftercare may include hospital admission, referral to community, psychological or social services, signposting services for self-referral, and/or discharge to general practice (3). However, patients have often described being discharged without a clear treatment plan in place and treatment failing to meet their needs (7, 11). There are limited services available with little flexibility in options for patients, with long waiting lists and exclusionary thresholds for referrals (12, 13). To address issues with mental health service provision for patients who self-harm, a small number of psychiatric liaison teams have initiated outpatient clinics to improve support for these patients, and there is preliminary evidence supporting the use of these approaches (14).

Over the past five years, there has been investment in the UK to develop and test interventions for self-harm in the ED context. This includes the SAFETEL safety planning intervention (15) (15) and programme grants funded by the National Institute for Health Research, the FRESHSTART project (also SAFEPIT) and the current Assured intervention.

The Assured intervention comprises key components of effective interventions for patients at risk of suicide (9): enhanced biopsychosocial assessment (16), safety planning (16–18) and follow-up contact (16–18).

The intervention was also informed by patients’ experiences of liaison psychiatry which has included lack of compassion and person-centred care (6, 19). In focus groups and semi-structured interviews, patients emphasized the shame and stigma they experience when attending the ED in crisis, and the need to feel understood and supported (7). They spoke about experiences of formulaic ‘tick-box’ assessments focused only on risk, and were positive about this new, more person-centred approach (7). Patients described how risk assessment questions could feel repetitive and like a checklist, which may not elicit true answers due to being too basic or may make the patient feel they have to answer in a certain way. They said that they would prefer to have a conversation with the practitioner, and to be listened to (7). Patients have also described the safety plans received from psychosocial assessments to often be limited to basic recommendations, that were not personalised to their own needs and experiences of what has been helpful (or unhelpful) in the past (20). The majority of patients are referred back to their GP after leaving the ED (21). Some are referred back to secondary care and some are referred to secondary care but may face long waiting times reflecting a gap in the system whereby patients could be better supported.

Drawing on international evidence (9), focus groups and semi-structured interviews with stakeholders (7), we adapted a brief intervention for people presenting to EDs for self-harm and suicidal ideation for the NHS context. The Assured intervention consisted of an enhanced biopsychosocial assessment in the ED to maximise the therapeutic potential of routine ED contacts, enhanced safety planning and solution-focused follow up sessions (22). There would be considerable potential for intervening at scale to reduce self-harm, as this intervention could be relevant to ~150,000 people who present to EDs in England with self-harm each year (8). A pilot study demonstrated the feasibility of carrying out a study in this context, training practitioners, obtaining consent from participants, delivering the intervention and collecting outcome data from participants (20).

We addressed the following research questions.

1. What were ED mental health liaison practitioners’ experiences of delivering and peoples’ experiences of receiving the Assured intervention?
2. What barriers and facilitators exist to implementing a brief psychological intervention for patients attending the ED with self-harm and/or suicidal ideation?
3. What are the mechanisms of the intervention that may lead to better patient experiences and outcomes?

## 2 Methods

A feasibility study of the Assured intervention for patients presenting to EDs with self-harm and/or suicidal ideation, when delivered by liaison psychiatry practitioners in four Emergency Departments in Southeast England. This was in preparation for a future randomized controlled trial to assess the clinical and cost effectiveness of the intervention in reducing re-attendance to the ED for self-harm and/or suicidal ideation.

Patients presenting to the ED are referred to the hospital liaison psychiatry team for a psychosocial assessment. Practitioners working in psychiatric liaison teams were trained to deliver the Assured intervention. Patients were invited to take part in the study in recruiting EDs if they met the inclusion criteria, which were that they were aged 16 years or over and had presented to an ED with self-harm and/or suicidal ideation. Self-harm was defined as an intentional act of self-poisoning or self-injury, irrespective of the motivation or apparent purpose of the act, and thus inclusive of self-harm with or without suicidal intent (3). Exclusion criteria were admission to a psychiatric hospital, cognitive or other psychiatric difficulties interfering with ability to participate, experiencing a psychotic episode, no capacity to provide written informed consent, needing an interpreter, Ministry of Justice patients subject to a restriction order and receiving intensive psychological input. A member of the liaison psychiatry team identified whether a patient was eligible for the study. If the patient met inclusion criteria, they were invited to take part, either by an on-site researcher or the practitioner. The researcher provided the patient with the Participant Information Sheet (PIS) and obtained written consent.

Participant allocation to a study arm was based on the practitioner they were allocated to, i.e., whether the practitioner was trained in the Assured approach (intervention arm) or was carrying out treatment as usual (TAU). In accordance with NICE guidelines, TAU involved a one-off meeting where a psychosocial assessment of needs and risks followed by discussion of care after discharge such as inpatient admission, referral to community services, signposting resources, or discharge back to primary care. Psychiatric liaison teams are given a 1 hour target to make contact with the patient after receiving a referral from the medical team in A&E and have a 4 hour target to assess and discharge. The standard psychosocial assessment (initial interview) usually takes 1 hour to complete and the Assured psychosocial assessment was designed to be completed in the same timeframe. Patients were often given a care plan summarising their discussion and a crisis plan with local crisis numbers.

Based on the principle of equipoise, patients were informed that practitioners had been trained in different ways of conducting assessments and follow-up care, and they would use the approach they have been trained in. Participating practitioners and patients in the intervention arm of the feasibility study were invited to take part in a semi-structured interview. The interviews were conducted by a Research Assistant, face-to-face, over the phone or Microsoft Teams, depending on participant preference. Patient interviews were conducted approximately 6 months after enrolment to parallel timelines for the feasibility study. 11 patient participants were interviewed after the 6-month assessment and one participant was interviewed 21 months after the 6-months assessment. Practitioner interviews were conducted at the end of their participation in the feasibility study. Interview was guided by semi-structured topic guides. Patient interviews explored patients’ experiences of receiving each component of the intervention and reflections on the therapeutic relationship with the practitioner. Practitioner interviews explored experiences of delivering each component of the intervention, experiences of delivering alongside their liaison psychiatry role, challenges faced working within the NHS context, and feedback on training. Patients received a £15 voucher as a thank you for taking part. Practitioners did not receive payment as interviews were conducted during their usual working hours.

### 2.1 The Assured intervention

The Assured intervention was designed to reduce repeat self-harm and/or suicidal ideation presentations to hospital and improve experiences of care and mental health.

The Assured intervention offers continuity of care as the patient is seen by the same practitioner after they leave the ED, maintaining the therapeutic alliance. This is at the heart of the intervention, as a trial of a previous intervention found that better therapeutic alliance was associated with fewer repeat suicide attempts at follow-up (23). This is consistent with evidence on the effectiveness of psychological therapies, where the therapeutic alliance is the strongest predictor of patient outcome and more important than the specific model of therapy (24, 25).

The intervention consisted of an enhanced biopsychosocial assessment and rapid follow up care.

#### 2.1.1 Enhanced biopsychosocial assessment

The biopsychosocial assessment conducted as part of the Assured intervention consisted of two components, a Narrative Interview, and a Safety Plan. Practitioners were trained to deliver carry out this assessment with an emphasis on specific therapeutic and principles techniques to maximise the therapeutic potential of the assessment:

##### 2.1.1.1 Narrative interview

The practitioner began the biopsychosocial assessment with a narrative interview that invited the person to tell their story leading up to the crisis. This process followed the principles of narrative interviewing from the Attempted Suicide Short Intervention Program (ASSIP) intervention (26). Training for the intervention entrusted practitioners to take a person-centred approach. Practitioners were trained to use open questions skilfully and consistently to ensure the patient decided the content that was discussed. The narrative interview also sought to develop a therapeutic alliance to engage patients and to help them feel hopeful and supported. The use of active listening skills, validation, and hope-instilling statements was covered in the training as means for achieving this. By opening the assessment in a patient-centred way, this sought to move away from a formulaic question-and-answer style assessment that has frequently been criticised by patients. Once the practitioner heard the person’s story, they then conducted their biopsychosocial assessment.

##### 2.1.1.2 Safety plan

At the end of their biopsychosocial assessment, the practitioner worked with the person to develop a personalised and enhanced safety plan, based on Stanley & Brown’s safety plan (27). The safety plan was co-produced to identify the person’s warning signs, internal and external coping strategies, informal and formal support to improve awareness and self-management of future self-harm. The practitioners were once again trained to use open questions with the aim of empowering the patient to draw upon their own expertise and skills to complete safety plan, rather than the practitioners. This safety plan also differed from the typical safety plan carried out in the practitioner usual role in that practitioners worked with the person to identify barriers to using these strategies, and subsequently steps to overcome said barriers, to maximise the potential for them using this in future crises.

#### 2.1.2 Follow up care

The Assured intervention provided rapid follow-up care after discharge from hospital. After discharge from hospital following a suicide attempt, most deaths from suicide occur within one or two weeks of discharge, often before the first post-discharge follow-up (28–30), emphasizing the need for follow-up within the days immediately following discharge (5). Rapid follow up care consisted of:

##### 2.1.2.1 Check-in phone call

The person received a check-in phone call within 72 hours of leaving the Emergency Department from the same practitioner.

##### 2.1.2.2 Follow-up sessions

The person was then offered follow-up sessions over a two-month period with the same practitioner using a solution-focused approach, at approximately 1, 4 and 8 weeks. The practitioner worked with the person to explore their future hopes and then identify the possible resources and strengths already present in achieving or working towards their future hopes (31). Focusing on the patient’s strengths and solutions rather than problems may help refocus the patient on the positives rather than negatives in their life and provide hope for the future (31). The solution-focused approach also emphasises patient empowerment, placing the patient in the role of the expert in the session. It encourages practitioners to trust that patients will focus on what matters most to them, both during the sessions itself as well as outside of each session - advising practitioners to strive to ‘leave no footprint’ on the patient’s change. Training for the follow-up sessions focused again on question techniques, such how to reframe questions in response to scenarios, and using scales to bring strengths and successes into the focus. For many practitioners, these principles required a shift from in their usual approach which involves the practitioner directing the interaction with a focus on assessing risk, formulating needs, and outlining actions and goals for the patient to follow.

##### 2.1.2.3 Letters

At three, six and nine months, the person received personalised letters from the practitioner to remind them of the safety plan and support networks.

### 2.2 Data analysis and coding

Interviews were carried out by five researchers and transcribed verbatim by researchers in the study team. The transcripts were analysed using a thematic analysis as described by Braun and Clarke, with an inductive approach (32).

Line by line coding was carried out by SH and NS. Both researchers initially coded 2 full transcripts out of 41 and reviewed and discussed approaches to ensure there was a consistent approach to coding. The remaining transcripts were coded either by NS or SH.

In a second stage, line by line codes were grouped into 32 categories. Initial categories were developed together in review by NS, RM and SH and iteratively revised as coding progressed. NS and SH both independently coded 15% of (152 of approximately 1000) line by line codes into categories, the rest of the coding was carried out either by NS or SH. There was 96.7% agreement between NS and SH, and a kappa coefficient of 0.744 (with a baseline kappa agreement of 87.1%). This represents a substantial agreement as per Landis and Koch (33).

Categories were then analysed together by SH and NS and developed into conceptual subthemes. NS, SH, and RM then analysed these themes to identify overarching themes which addressed the key research questions.

## 3 Results

### 3.1 Participants

Of the 16 practitioners who delivered the intervention, 13 practitioners took part in a qualitative interview. Of those, 12 delivered the intervention and one withdrew from the study prior to delivering the intervention but agreed to take part in an interview. Practitioners were an average age of 43 (SD =6.16), 62% were female. The majority (77%) were psychiatric liaison nurses, and the rest were psychologists (15%) or doctors (8%).

In the feasibility study, 46 people were assigned to the intervention arm. Of the 46 patients in the intervention arm, 28 (61%) attended at least one of the three follow-up sessions. 20 patients attended all three sessions (44%), two patients attended two sessions (4%), six patients attended one session (13%) and 18 patients attended none of the offered sessions (39%). All were invited to take part in an interview about their experiences and 27 took part in an interview. They had an average age of 30 (SD = 12.78), 70% were female and 56% were White British. 11 patients (41%) attended all three sessions, three patients (11%) attended two sessions, four patients (15%) attended one session and nine patients (33%) attended none of the offered sessions. Carers were also invited to take part where they had been involved in the intervention, but in practice carers were not typically involved so only one carer participated in an interview.

Patient interviews were conducted between 23/06/2020 and 17/06/2022. Practitioner interviews were conducted between 15/07/2020 and 28/03/2022. As the intervention took place during the COVID pandemic, mode of delivery, whether virtual or in person, was flexible according to the wider situation and taking account needs of the service and patient. The psychosocial assessment was conducted in A&E. Follow-up appointments were conducted online or in-person in a room within the hospital but that was not the initial assessment room. Letters were mainly sent in the post, however some practitioners mentioned emailing them due to patient preference.

### 3.2 Thematic analysis

Overall, five overarching themes were identified.

**Theme 1: The intervention mostly gives agency and hope to highly distressed patients, and helps patients implement strategies to support their mental health; however potential adverse effects were patients becoming overwhelmed or triggered which require mitigation.**

Patients in crisis described feeling distressed, overwhelmed, and finding it difficult to engage at the initial assessment. Many mentioned struggling to think clearly as under the influence of substances and not remembering what was discussed at assessment.

*‘When I go home I don’t remember what’s happened, I don’t remember what the care plan would have been erm it’s very hard like … I have to look through my own notes to be able to know actually this is what was kind of discussed in a meeting or that’s what the next follow-up would be. So, perhaps maybe not speak to a patient until they’re fully like erm I would say back in their senses to a point where they’re able to remember and recall a conversation’ (patient, age range 20-30)*

Practitioners and patients conveyed that intervening soon after the initial crisis, once people start feeling more stable, provided an opportune window for constructive work. This time was best for providing help when it was needed, and reflecting and addressing how to prevent future crises. Practitioners noted that when patients started feeling better and recovering from the crisis state they sometimes did not want to think back to difficult times, highlighting the need to engage them at the right moment.

*‘being able to have someone to speak to after that as a follow-up is super important. Um, because like I completely get how stretched the NHS and health services are, but it is hard, you know, if you come in when you’re at your lowest and there’s no kind of follow-up, or a long wait, that kind of thing. So it was good that I had someone fairly immediate to follow-up on.’ (patient, age range 20-30)*

Both practitioners and patients emphasized the need for a gentle and sensitive approach to engage these patients who may feel guarded about opening up, and not to introduce too much pressure at assessment.

*‘[by follow-up] whatever she said, like, if she was like, you know, not being really caring with me or, like, not, erm, not tailoring her questions towards me, it didn’t affect me negatively because I was fine by that time. But if [practitioner’s name] had come to speak to me before the ambulance arrived when I was, erm, at home, that-that probably would’ve been a- a stickier situation. I would’ve just, you know, what are you talking about, like, I would’ve said something like that to her.’ (patient, age range 20-30)*

Patients commonly described feeling isolated and unsupported in their lives generally, either due to few or difficult close relationships. Thinking about the future rather than current circumstances as part of solution-focused approach was a challenge and something new for many patients.

*‘I feel like my life is quite chaotic, every day at the moment so I feel like trying to talk about stuff in the future seems like very wishful thinking.’ (patient, age range 20-30)*

Similarly, practitioners felt patients were often stuck in a negative space or focused on their idea of what was wrong, making it a challenge to guide a shift in thinking.

*‘[the intervention is about] imagining a better future and [I had to make] that gel with what she was talking about [which] was to do with how she relates to other people and the problems other people give her, you know … I couldn’t really wrestle in my mind how to get her into that box [of imagining a better future]’ (practitioner, age unknown)*

Despite this, patients did report finding the intervention helpful. Patients described how the intervention gave them both hope that things would improve and strategies to put in place. This gave them the agency to improve their mental health, manage and prevent future crises.

*‘she gave me, you know, not false hope, that everything suddenly will change. But she gave me like a tool that I can you know, I can … I’m resilient enough to go and you know, continue to fight for my health. And so yeah, she gave me this tool that it’s really, really, really positive.’ (patient, age range 40-50)*

Patients described it being helpful to involved family but only if they felt supportive, for some this would add unhelpful pressure or make the space feel less safe, stopping them from fully opening up. The one carer who was interviewed described how anxiety-provoking it was not knowing what was going on for their relative. They expressed a keen desire to be involved and to support and help the patient in managing their mental health after the crisis presentation.

There were some potential adverse effects identified.

A couple of patients described feeling overwhelmed when asked to think about the future as part of the solution-focused approach. One became too busy employing all the strategies suggested in the safety plan to the point that this started to deplete them, needing to then review how to manage their time and energy within the follow-up.

*‘the first follow-up session we had come up with a plan of what I would do to get out of like these, this really depressive mode, I would be more social I would do this I would do that, I’d find a hobby, but then I’d go on the other extreme and that was my last and final follow up where I’m so social I don’t wanna go home I don’t ever wanna be at home I wanna spend a lot, I wanna, I’m finding so many hobbies that I’m not able to actually find a balance in things, so then we had to come up with a plan for trying to keep a bit of a balance for that and it’s like you know I’m not sure how great this whole plan thing works, but definitely having interaction with someone does make a difference.’ (patient, age range 20-30)*

Furthermore, both patients and practitioners frequently referred to ‘goals’ in their interviews: this is not recommended as part of a solution-focused approach (although it may be usual practice for practitioners) as it may put additional pressure on patients to achieve what may be unrealistic expectations and may become overwhelming.

Some patients described being triggered emotionally by the conversations. They wanted additional support or strategies to help calm them after conversations, or for the practitioner to understand the need to end on ‘a lighter note’. For some this seemed to be a problem largely related to a less sensitive approach employed by the practitioner. The risk of re-traumatisation was a key concern raised by practitioners implementing the intervention. Some described hesitating to explore in depth what led up to the crisis when they first met the patient for this reason, and wanting to focus more on the present situation. Others feared that by following up they would be reminding the patient of the crisis in an unhelpful way.

*‘When I did speak with [the practitioner] it did trigger me and afterwards I didn’t have anything to do. I feel like mindfulness at the end of sessions would be helpful or something like that to bring me back to the present moment and then focusing on what you’re going to do after and do you have plans, are you going to eat food and stuff like that.’ (patient, age under 20yrs)*

Patients and practitioners highlighted the limitations of how in depth this short intervention could be, and whether the impact could be consolidated enough to have lasting effects. Others highlighted that the intervention could not impact on patients’ external circumstances such as housing and finances which may be a significant determinant of patients’ suicidal ideation. Some practitioners felt that in later follow-up sessions, the addition of further complementary techniques to solution-focussed work such as behavioural activation or DBT based on patient’s needs, would be helpful and more relevant to the patient’s situation.

**Theme 2: The intervention works by creating a space for patients to express what is important to them; facilitating insight and reflection, validating emotions, and building self-esteem, helping patients manage boundaries and trial what works to support their mental health.**

A key aspect described in relation to the success of the intervention was the fact that the intervention facilitates a patient-led and patient-centred approach: the intervention allows patients to bring what is important to them and have focused input on that rather than feel that the practitioner has applied a generic approach to them.

*[I mentioned] that I really enjoyed creating the garden, and [the practitioner said] it sounds like you’re a really creative person and that helps you a lot. So, I think it was sort of about using skills I knew, not that I knew I had, but just developing them further and having the confidence to do them as well, and the motivation.’ (patient, age range 50-60)*

Data suggested the following key mechanisms of change when the intervention was applied:

• Facilitating reflection, guided by practitioner

*‘I think it was the whole like being able to have reflective conversations with myself, if something wasn’t working out it was about having realistic conversations with myself, reassuring myself not setting myself these crazy targets that’s not achievable in the perfect way that I expect, um so yeah I really do think it’s about being able to understand myself and have those thought provoking conversations with myself … [the practitioner] did kind of like push me to think about these things which then enabled me to do those on my own. Sometimes you’re just so caught up in what you’re thinking of you can’t think anything bigger until someone kind of questions you a little bit so that then you end up questioning yourself, and you’re able to do these things’ (patient, age range 20-30)*

• Enabling catharsis and reducing loneliness through having someone to listen and talk to

*‘it’s getting things off your chest. It’s basically telling someone … Y’know, at the time I had no one to talk to’ (patient, age range 30-40)*

• Helping normalise and validating emotions

*‘so like the feelings that I was feeling a lot of the time I didn’t know if they were normal or if it was okay to feel like this or okay to have a reaction or an emotional reaction to a situation um a lot of the time in my setting, I’m told that I’m crazy and it’s not normal or whatever it is so I was able to just ask like, is this normal? Is it me? You know, um, and [the practitioner] was able to actually help me realise what wasn’t normal in terms of other peoples’ behaviour’ (patient, age range 20-30)*

• Helping patient to manage emotions and boundaries

*‘[As a result of the intervention I am] prioritising things, and accepting that I’m not human and I can’t do everything,’ (patient, age range 50-60)*

• Enhancing patients’ awareness of what is available and what they can do to support mental health

*‘We went through a cycle of what we wanted, what I wanted to achieve and what, so sort of who I could talk to, who would be – not goals but what was the best way of getting out of the, out of my own head if you know what I mean and so we done a sheet I believe, I think it was at the time we had the bubble work of who, I talk to friends and would family help and having family around would that help and maybe speaking to an outside source of people you know like Samaritans, would that help me.’ (patient, age range 30-40)*

• Enabling patients to trial out different strategies within a safe space and review with practitioner

*‘We usually talked about how I’d been since I had last seen her and then sort of say, how, how that related to the crisis plan. Which points did I manage to use off of it? Which ones didn’t I use? And like, is that because they weren’t useful or I didn’t go through it myself like and I think a good example will probably be like we came up with a list of people that I would reach out to and we had a big conversation about why I don’t feel comfortable reaching out and why I delay that as much as possible even though I know there’s people who do want to help me. And sort of the steps that I have to take to help me get to a place where I do feel like I can reach out … so it was almost like we try Trial-and-errored parts of the safety plan to see how it worked in practice as opposed to just in theory.’ (patient, age range 20-30)*

• Providing positive feedback and encouragement, enhancing patients’ self-esteem and awareness of strengths

*‘when somebody point something out to you and then you suddenly began noticing it all the time when you hadn’t thought about that before just seeing sort of like these small steps and goals in my day-to-day life. Whereas previously even though they were there I just hadn’t thought about them … noticing when I was able to trust or share with people around me. Like, I didn’t, like conversations I didn’t like having or didn’t want to have about my mental health and that they weren’t a complete disaster like, I always thought they would be. So as part of that, we spoke a lot about small victories and yeah, like acknowledging the progress I was making along the way’ (patient, age range 20-30)*

**Theme 3: The intervention re-imagines the practitioner-patient relationship and facilitates trust and new possibilities for patients who have difficult relationship and help-seeking histories**

Patients described existing disillusionment with support offered, feeling shamed, judged, dismissed, and not listened to by professionals and how they felt treated differently in the intervention.

*[In the Assured intervention} I wasn’t you know, judged, like I am by other professionals, that they basically, I feel that they condemned my decision’ (patient, age range 40-50)*

Patients described how, when in crisis, they are extra sensitive to how they are treated, with caring approached being particularly beneficial or healing and insensitive approaches or actions having potential to cause greater distress and disillusionment, as patients had often experienced in the past.

*‘I called my GP up and said I want to die I want to kill myself and they gave me a number to call to speak to a crisis team and the crisis team was on training that day so I had no one to speak to at that point and I wasn’t going to ring my doctor back up again going they’re not on they’re on training … at that point I was like no one really cares I want to die’ (patient, age under 20)*

Practitioners described an existing culture of feeling like they are battling rather than working with patients, which is not conducive to a softer more attuned approach.

*‘you do get jaded and you just think that everything’s going to be a battle, er, in A&E, so [the intervention has got me] maybe thinking more about how to do less warfare and how to be more conciliatory, maybe, in A&E, than, than I’ve been before … because as I say, you’re expecting a fight, and so you go looking for a fight almost (haha)’ (practitioner, age unknown)*

The interviews provided evidence that the intervention could help shift the existing dysfunctional dynamic and foster a more caring and collaborative relationship between patient and practitioner.

In particular, patients highlighted how the intervention felt different from usual care. They valued the more informal, conversational approach. Many described feeling calmer and more at ease than with usual assessments.

*‘She introduced a very relaxed and welcoming environment. A very safe environment where I felt like I could speak to her and everything-, anything I was saying she wasn’t questioning or judging. She wasn’t even just being silent, you know, she had something to say as well, which was nice. It was like a conversation rather than her sat there taking notes and me just talking.*’ *(patient, age range 20-30)*

Patients also described how they felt the interaction was more human and personal rather a professional-patient relationship.

*‘she was more like a human being than the professional, so she asked the questions, really simple and to the point, without you know, any … any elaboration or any you know, difficult language for me to understand. So it was … it was really you know, like human being, talking to a human being.’ (patient, age range 40-50)*

Some highlighted that it was helpful for the practitioner to be frank about what they could and could not help with.

Many patients spoke about how they felt the practitioner genuinely cared and how the intervention opened up a space where they could build a connection with and work with the practitioner.

*‘it was the first therapist that I’ve felt – I feel like I’m quite open and I don’t mind talking about this kind of stuff. But it’s nicer when you feel like a connection with that person – you feel like they do actually care.’ (patient, age range 20-30)*

Practitioners and patients described a joint exploration of the patient’s journey and joint creation of plans together. The give and take between practitioner and patient opened up a two-way learning process which was respectful of the patient’s knowledge and experience.

*‘I think it was more like as we were going through and coming up with ideas, she’d suggest things and I could be completely honest like, “No, I know that’s not going to work.” She won’t be like “oh maybe you should try it”, she trusted that I knew. So, for example, like, I think she did have a list of various distraction techniques in front of her, so we went through those and there’d be some ones where I’d be like “yeah, no, I tried that, and I know that’s not going to help at all”.’ (patient, age range 20-30)*

This seemed to additionally inspire patients and increase investment in the intervention.

*‘It’s like I see that someone really can help me, so I open up and I try to do everything you know, positive, to find anything positive you know, and go that way. So it’s like you know that you know, the professional wants to help you, so you do everything you know, to help yourself first.’ (patient, age range 40-50)*

The practitioners also gained motivation from patient engagement.

*‘[patient] was so enthusiastic he was the most enthusiastic person I’ve ever met; he was so psychologically minded he’s like I need this I really need this I really want to do it I really want it to work and he was so interested in every aspect of the intervention and I think that really motivated me to make it want to work for him as well- We definitely bounced off each other’ (practitioner, age range 40-50)*

Both patients and practitioners reported the importance of non-verbal contact for enabling good communication, expressing emotions, and building alliance, which was more difficult when on the phone or online than in person. Some also described more difficulty in opening up and having a more in-depth conversation when on the phone.

*‘it’s a lot harder to hide something when face to face than it is on the phone because I can say whatever I want on the phone … Whereas in terms of face to face, you’d be able to see if someone, like, I think you’d be able to see the pain that someone’s in or they’re erm, or whether or not they’re about to I suppose have a tearful moment and yeah, because you can see how the mood changes, can’t you?’ (patient, age range 30-40)*

**Theme 4: The intervention requires a significant shift in professional attitudes, culture, and way of working, moving from a reactive to a more proactive role, that needs support to facilitate but is rewarding**

For practitioners, the intervention involved letting go of their expertise and formulations and learning new skills as for some this was a significant shift in the way they worked. Practitioners were instructed not to set explicit goals in sessions, and instead help the patient through their choice of questions to unearth their own answers and actions, and trust that they would action those which they chose to be most important to them. This required practitioners to be vulnerable, and to relinquish some control, which was somewhat disconcerting – one practitioner mentioned that the intervention felt like alchemy in how it worked.

*‘it’s quite nebulous to kind of think about, you know? It made a lot of sense when [Supervisor] said it but I [laughs], I’m trying to remember that, channel that back later on is quite difficult … just what you’re trying to actually achieve, or the process because it sort of seems like alchemy’ (practitioner, age unknown)*

Supervisors mentioned the importance of more pastoral support to help practitioners make the transition

*‘once a week [we had a] kind of a group catch up. How was everything going? more along the lines of pep talk, motivation, well-being kind of things, and then the ones [to] ones were more clinical um, specific if this makes sense.’ (practitioner, age range 40-50)*

Practitioners felt that, given the looser structure of the intervention, they needed to be able to practice and be mentored in order to develop confidence and skills and a sense of competence in application.

*‘had there been somebody with a bit more solution focussed therapy experience on the call as well, who would have been able to sort of redirect or kind of chip in if I was really struggling, then that might have been, that could have been really helpful’ (practitioner, age range 30-40)*

Some described reverting to other approaches when the intervention technique was a challenge. A focus on goals was often mentioned despite this being discouraged in the intervention. This aligns with evidence in theme 1 of patients feeling overwhelmed and becoming too busy. A difficulty faced by practitioners is that skilful and subtle language use, which is important when delivering the solution-focussed approach, may time before it can be mastered.

*‘I think I kind of veered away from it sometimes, cos I think it’s quite difficult to break away from what you normally do, doing something completely different, and I think with time, that kind of takes time and repetitiveness and continuity.’ (practitioner, age range 40-50)*

Practitioners described their existing role as often having to react to emergencies and focus on risk reduction, doing what is needed in the moment rather than thinking long term. The intervention requires practitioners to step back from a primary focus on risk reduction and switch to promoting mental health, whilst still having to hold clinical responsibility for risk, which is a challenge.

*‘I think that’s a major thing that this whole process this whole intervention has taught me to really explore self-harm and suicidal thoughts because what we, what often happens in an A&E setting where you have patients that frequently attend with self-harm you stop asking those questions it becomes, self-harm becomes a generic term. And it’s not generic it’s individual to each person the intention behind it is different and it can be different each time if you’ve seen even if you’ve seen the same person it can be different each time it could be triggered by something different it could be more severe it can even change people’s type of self-harm can change as well people can go from biting or headbanging to cutting or burning and we have to look at those changes and think about why has it changed and what does this indicate about the person’s level of risk’ (practitioner, age range 40-50)*

Both patients and practitioners found the opportunity to have a consistent, longer space to work together rewarding.

*[I valued the consistent practitioner because] I always find that if you’re getting different people every single time, they don’t know what you said before, they don’t know your history and it-, always find then you’re repeating yourself on the first one so you’re not actually progressing anywhere’ (patient, age range 50-60)*

Practitioners described applying the intervention was a positive experience:

*‘I’ve really enjoyed using the intervention.’ (practitioner, age range 40-50)*

For practitioners there was the added benefit of getting feedback on what happened to the patient which they could then use to improve their practice

*‘with a single point of contact that we might have with someone, it sometimes it feels like you don’t know what happened with that patient and you kind of feel there’s no sense of closure*.*it’s actually quite beneficial and rewarding for the clinician to actually get the opportunity to see how someone is feeling after things have settled down or how they are getting on afterwards really ‘ (practitioner, age range 30-40)*

A key challenge to facilitating this intervention is the ED environment. The busy environment, with multiple competing demands and rigid, antisocial shift patterns, made it a challenge for practitioners to find the space for this without significant support from leadership. Being unreliable for appointments also risks damaging the practitioner-patient relationship and making it likely that patients will disengage without completing the intervention. Missed interactions were reported by patients who did not complete the full intervention,

*‘in our team in ED setting it’s really difficult. I feel slightly protected because of this, you know senior post, so I kind of, I have the luxury to block my diary for one morning or whatever and then say look I’m not available to do ED or … but again, if it’s really busy I just obviously need to go and see the - whatever is needed. But if they don’t have an allocated time protected time for follow ups. I think that, I think is also something that we need to reflect, to make it better, um, because potentially some reasons of DNA was, it was agreed with the patient ‘I’ll call you on Friday at, in the morning’. And then I didn’t, and I’ll do it Friday afternoon and the patient was like ‘No’’ (practitioner, age rang 40-50)*

The physical space, being noisy, bright, and clinical with patients waiting in general ED waiting rooms and often without dedicated consulting rooms for the intervention also was not conducive to a calm and supportive therapeutic conversation.

Data pointed to the practitioners holding preconceived ideas about what works with certain patient groups and finding it difficult to let go of established assumptions (that may be cultural rather than evidence based). This included feeling like those with personality disorders and teenagers and would be unable to benefit from the intervention and applying it with the former would be particularly risky.

*‘I would always think to myself, er, you know, is this person that, is this the person that I want, in anyway attached to me, with what I know what they get up to in the community [laughs] some people say no, they’re under a team for them to deal with, so no, let them carry on, er, in that, you know, the very severe kind of PDs and stuff that, you know, you don’t want any kind of attachment to. Especially if you’re going to, especially in a, a, er, a study kind of environment where you’re going to be doing something that’s experimental. With some people you don’t want to experiment with [laughs].’ (practitioner, age unknown)*

This was also seen to affect recruitment to the intervention, with implicit agreement not to recruit some who met criteria for intervention as they felt they would not engage.

*‘one of the things that struck me is that of course you’re sort of self-selecting the people that you’re going to be doing this with … if they met the criteria and the people, just take the people as you found them…… but it was kind of a running joke in the office to some people who said Well why don’t you take her on she fits the criteria and everyone laughs [laughs] and goes no chance.’ (practitioner, age unknown)*

Some patients who did not fully complete intervention described feeling the practitioner’s input was not attuned to their needs.

*‘It was just like they didn’t really ask enough about me, but they were asking stuff about everyone else. And it’s like, I came there for me.’ (patient, age under 20)*

Others felt that the practitioner lacked empathy.

‘*it wasn’t very sympathetic, and it was more like making out, like, why have you done this? Like you’re stupid sort of thing.’ (patient age range 20-30)*

This highlights the difficulty in shifting negative dynamics and how not all trained practitioners were able to effectively apply the principles of the Assured intervention.

**Theme 5: Disrupting the limitations of point-of-crisis care by opening up a new and timely therapeutic space and holding and supporting patients to access further support.**

Patients in crisis described there being a dearth of support available for them, with services being hard to get into with long waiting list for more in depth supports. Some reported repeated rejections or being disadvantaged through moving homes and having to be re-referred.

*‘health professionals, they don’t want to really help, they just you know, it’s … nothing really changed, they just pushed me to other people, and the other people pushed me to other people. And this is how I end up in vicious circle, nothing changed’ (patient, age range 40-50)*

The intervention thus provides an additional capacity for the mental health system to support these patients for a longer period post crisis alongside developing a more therapeutic relationship rather than pure crisis care (See Theme 3), and helps them to feel kept in mind by professionals.

*‘she was always really clear that there were places I could reach out to, or if I if I needed something in between. So it wasn’t yeah, so I wasn’t worried that I wouldn’t have anybody to check in for the next 6 weeks or however long if something went wrong.’ (patient, age range 20-30)*

The intervention acted as a conduit to patients accessing further care in multiple ways:

• Practitioners making referrals to further services

• Practitioners helping follow up referrals when they did not go smoothly helping keep patients in the system

*‘[the intervention] enabled me to follow up, she kind of got lost a little bit and I made a referral with the expectation she would get onto the STEPS programme. I looked at her notes the other day and she’s kind of engaged with services, which is nice, and I think that’s what she’d wanted. And then I think the process of being involved with the Assured study helped me to be in the right time. You know, in the right place, at the at the right time, in a sense, to make that happen.’ (practitioner, age range 40-50)*

• Providing a buffer and holding space for patients who are waiting for further care

• Providing an initial positive introductory intervention for patients who may then be more likely to take up further care

*‘[the patient was concerned] a therapeutic contact would open things up too much for her but she appreciated the solution-focussed approach because it meant that she could open up but in a way that it was safe for her’ (practitioner, age unknown)*

• Increases patient trust that the mental health care system can support them and thus reduces the likelihood of patients disengaging

*‘not only does it reassure me as the clinician but I think it reassures them, the service user, on the point that there are all these steps before we get to, you know, before we get to where you can’t tolerate your stresses and there is nothing for you at that point.’ (practitioner, age unknown).*

There were some logistical barriers to the intervention. As patients often came from quite far it was difficult for them to travel to the ED for face to face follow up. Many reported that they were more likely to attend because of the option of phone or video follow up, and many also preferred the flexibility of follow ups in the evening or weekends, whilst some found the practitioners’ availability difficult to work with. Some patients who did not complete the intervention described not getting follow up calls or not being able to follow up if they missed the practitioner’s calls. Most patients reported not receiving or not remembering letters and so we were not able to fully analyse impact of the follow up care component of the intervention: this is likely to have been partially due to the timing of the interviews being before full follow up was completed, but also may point to lack of effective delivery or lack of impact of this component of the intervention.

The intervention has the potential to complement other parts of the system. Practitioners described the potential for shared notes with other services already present in the system and the possibility for safety plans to be carried with the patient and used by other services. However, practitioners described struggling with the visibility and clarity of what the intervention was, and being concerned that patients would be more likely to be rejected by other services if they saw they were receiving the Assured intervention, thus causing them to not write in shared notes or communicate with other services.

*‘I am very limited in what I can do for them. I am not a service, like… … because she got a letter from us to give to her Uni saying that she is under this follow-up service but we’re not really anything, you know what I mean. But her university got the impression that she was seeing someone and she was getting some help so she was okay. But we’re not necessarily the people for that. She should be going through a community mental health team and seeking support and getting a proper assessment.’ (practitioner, age unknown)*

Some patients also reported not wanting to engage with the intervention as they were already engaged with services or receiving other crisis support. Practitioners also highlighted the need to be very clear about what the intervention was and when it would end with patients who may easily feel abandoned or let down. For many patients this was the beginning of further care, others wanted more of what had been a helpful and supportive intervention. Some patients accepted the limited nature of the intervention given resources, and others felt it had got them where they wanted to be. Others reported feeling anxious about being left alone after the intervention and found it reassuring to know they had options for support.

*‘As long as somebody had talked me through it, yeah, and kind of explained the steps for me as opposed to kind of just being like, you’ve got your three sessions here and then afterwards it’s up to you to sort out yourself. I think I would’ve found that really difficult’ (patient, age range 20-30).*

## 4 Discussion

### 4.1 Key findings

We investigated practitioners and peoples’ experiences of a brief psychological intervention following ED presentation for self-harm and/or suicidal ideation. We found that the intervention gives agency and hope to highly distressed patients, and helps patients implement strategies to support mental health. However, there are potential adverse impacts of patents becoming overwhelmed or triggered which require mitigation.

We sought to understand the mechanisms by which the interventions create better patient experiences and outcomes. We found that the intervention works by creating a space where the patient can lead with what is important to them with personalised feedback from the practitioner; facilitating insight and reflection, validating emotions, and building self-esteem, helping patients manage boundaries and trial what works to support their mental health. Additionally, the intervention re-imagines the practitioner-patient relationship and facilitates trust and new possibilities for patients who have difficult relationship and help-seeking histories.

We sought to understand the barriers and facilitators to implementing a brief psychological intervention for patients attending the ED with self-harm and/or suicidal ideation in the UK mental health system. We found that the intervention requires a significant shift in professional culture and way of working practically by doing follow-up work, moving from a reactive to a more proactive role. This needs support and supervision but is rewarding for practitioners. Practitioners struggled with this new role which required them to step back from risk management and formulation and work more openly with the clients, thus disrupting existing power dynamics. Practitioners needed support from managers and through supervision to find the physical and mental time and space to build confidence and to allow them to create a longer-term relationship with the patient.

**Figure 1:**
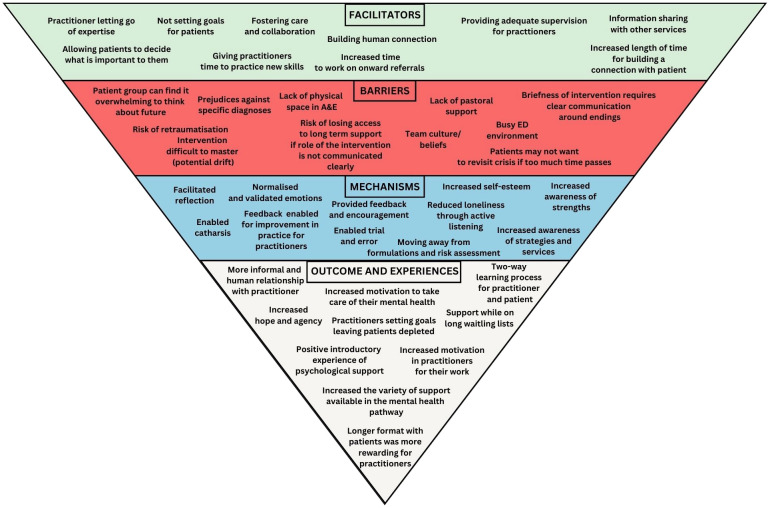
Experiences of delivering and receiving the Assured brief psychological intervention for people presenting to emergency departments with self harm - summary of key findings.

We also found that the intervention challenges the limitations of point-of-crisis care by opening up a new and timely therapeutic space and holding and supporting patients to access further support. There is no pre-existing infrastructure in EDs for follow up contact. It was clear the existing ED environment, with the lack of privacy and protected space, a scope of work that is reactive to what emergencies come in through the door day to day, and multiple competing demands, does not support this. There would need to be some restructuring of how the liaison services work, including review of physical space available for follow up work, to accommodate the intervention. The format of sessions is also important for engagement, many patients found it easier to open up when face to face, but remote sessions and flexible timings made the intervention more accessible. Logistical issues and missed contacts or broken promises contributed to disengagement, as well as persistence of unhelpful communication and attitudes from practitioners despite the training. In relation to the wider mental healthcare system, there were concerns about adequately communicating responsibilities and boundaries between different services, and ensuring the intervention was seen as complementary rather than an alternative to existing mental health services. See **Figure 1** for a visualisation of key findings.

### 4.2 Comparison to other literature

This analysis describes the acceptability and impact of providing the Assured intervention, a brief psychological intervention with rapid follow-up for people presenting to the ED with self-harm/suicidal ideation, in a UK NHS context. Rapid follow-up is critical given the increased risk of completed suicide in the 3 months after ED attendance or admission (34). Our paper builds upon a small but favourable previous international literature that suggests brief interventions can be effectively delivered in ED to reduce suicidality or depression (9, 35) and recent UK studies suggesting it is feasible to deliver safety planning with follow up interventions in the NHS ED context (15).

Our analysis identified a number of mechanisms of change: a different kind of practitioner-patient relationship; facilitating reflection and insight around issues and triggers related to self-harm behaviour; understanding and accepting emotions; feeling validated; giving patients more practical skills of managing boundaries; strategies to support mental health; providing a space for new ways of thinking/behaving; feedback and positive encouragement. This aligns with international evidence showing that effective interventions have included components such as increased understanding of suicidal behaviour; patients feeling seen and heard by professionals; helping people to become aware of problems/vulnerability/events linked to the behaviour; exploring ambivalence and motivating people to engage in safety planning and help-seeking and problem solving, developing practical strategies to manage future suicidal crises along with signposting to helplines/professionals (9). These findings underline the role of the therapeutic relationship and validation a two way learning process between patient and practitioner, and positive, personalised feedback in facilitating patient improvement (36).

This work extends previous research on the experience of those attending with self-harm and/or suicidal ideation in the emergency department which has described existing failures of the system Practitioners become hardened to patients and risk assessments address staff fear more than patient need. Patients in turn fee unsupported and judged, not receiving an accepting and human connection which offers hope when life feels hopeless (6, 7, 13, 37, 38). Our analysis shows the potential of the Assured intervention to re-imagine crisis care and alter the unsupportive dynamics of the patient-practitioner relationship.

Recent critique of psychiatric care highlighted the presence of a defensive and exclusionary culture within services. Beale highlights this as a systemic issue, where risk aversion and lack of resource may drain its clinicians of compassion, losing sight of the human being behind each ‘protected’ bed and rejected referral, and prioritise professional accountability over keeping the patient supported (39). Patients with complex emotional needs who may turn up repeatedly with similar issues and without either getting better or dying are particularly vulnerable to being seen as a problem within this system is not supported and resourced to accommodate them (39, 40). These factors are reflected in the practitioner’s concerns that patients taking part in Assured might be excluded from further care from other services. Despite these barriers, it was clear that, as part of the intervention more useful and hopeful conversations could be had between practitioner and patient, suggesting its role in enhancing capacity to deliver compassionate care and creating a capacity for care in the system. However, our analysis suggested some practitioners held fixed views about certain patients and their ability to benefit from the intervention which may have impacted the engagement. Previous literature has highlighted the stress patients feel when faced with bias from ED professionals that may align with negative internal self-conceptions or feelings about their self-harm and suicidality (41).

This paper contributes to a growing literature on the effective application of solution-focussed approaches in mental health settings (42, 43). Our findings build on work that suggests solution focussed interventions can instil hope in suicidal patients (44); and that found solution focussed training for nurses could improve could improve confidence and competence in delivering mental health promotion to and addressing the psychosocial needs of patients; confirming the positive impact this can have on patients themselves (45, 46).

The fact that practitioners reported seeing the value in the approach and found it rewarding and enjoyable to carry out the intervention is particularly important given job satisfaction is a core component of burnout and poor retention within this psychiatric nursing (47). The importance of supervisory spaces for both wellbeing and technical support is highlighted here, and may also be fruitful spaces where negative culture that damage practitioner-patient relationships can be explored and restructured.

This analysis highlighted significant systemic barriers to delivering the brief intervention in ED. Alongside existing literature that highlights an overstimulating physical environment that lacks privacy (41), this paper adds detail in regards to nature of liaison work being reactive to the environment and prioritising in the moment, with rigid shift patterns that do not facilitate a planned and prolonged therapeutic contact. This is particularly important given the findings both in this pilot and in previous literature regarding the value of a consistent relationship with a practitioner (17, 22).

### 4.3 Strengths and limitations

Strengths of this analysis are the combination of views from patients and practitioners on how that intervention is implemented in practice and key mechanisms of change across patients, practitioners, and the system. 59% of patients who took part in the session were interviewed, a range of patients who did and did not complete the intervention were included which reduced the risk of bias towards opinions of those who found the intervention more favourable. However, it is unclear how representative these views are of those who chose not to participate in the feasibility study, not to be interviewed or could not be contacted. The generalisability of these results to other healthcare systems is unclear.

### 4.4 Clinical implications

The results of this suggest implementation of a brief intervention for self-harm and/or suicidal ideation in the ED requires significant change in staff and mental health systems. Our findings highlight that patients feel unsupported and left to linger on waiting lists and that there is a gap in the offer between crisis presentation and longer term follow up care, which the intervention can fill. Our findings also suggest that the intervention supports the connection of patients to further care and increasing trust in the system. A compassionate and non-judgemental approach is highly valued by patients who are particularly sensitive to how they are treated when in crisis. Patients commonly spoke about the importance of feeling validated by professionals rather than being given the message that they are a burden on the system.

We make the following recommendations to ensure successful implementation of a brief psychological intervention for self-harm and/or suicidal ideation in ED:

1. Support from management and leadership to facilitate staff development time and flexibility to deliver intervention.
2. Communicating and making visible the role of the intervention as complementary and additional rather than an alternative to usual care to other services in the system
3. Specific supplementary training or supervision for practitioners around mitigation of adverse impacts of patients feeling overwhelmed or triggered by conversations.

We make the following recommendations for mental health crisis care outside of the intervention itself:

1. Supervision and team meetings should focus on reflection and reworking of unhelpful beliefs and hardened attitudes towards patients.
2. Practitioners’ training and development offers should include narrative interview, personalised safety planning and solution focussed approaches which our and previous research suggest help positively alter the professional-patient interactions.
3. Practitioners should be made aware of the therapeutic value of a compassionate and non-judgemental approach.
4. Mental health care systems should review pathways and provision to assess and strengthen support post crisis presentation.

### 4.5 Research implications

Results indicate that a randomised controlled trial testing the clinical and cost effectiveness of the Assured intervention is warranted. Additional findings in relation to the potential of the approach to re-imagine new therapeutic practitioner-patient relationships and enhance capacity of the mental healthcare system to care for patients warrant further investigation. Future research should also investigate the impact of evidence-based brief psychological interventions for self-harm and/or suicidal ideation in ED in other healthcare systems.

## 5 Conclusion

This study suggests that a brief intervention for suicide and/or self-harm in the ED can create new capacity in the mental health system, linking patients to appropriate further care; reimagine the practitioner-patient relationship to one that is more human, personal, and collaborative; and address the need for both hope and targeted strategies that give patients agency to support their mental health. The study demonstrates the acceptability of the Assured intervention (which contains evidence-based components of enhanced psychosocial intervention with a narrative interview, safety planning and rapid structured follow up with a consistent practitioner using a solution-focused approach) in the ED context, but that this change in ways of working requires clinical, organisational, and systemic support. The study and complements further findings on feasibility of the intervention that support the case for a full-scale randomised controlled trial to assess the clinical and cost effectiveness of the Assured intervention (20).

## Data availability statement

The raw data supporting the conclusions of this article will be made available on request.

## Ethics statement

The studies involving humans were approved by London-Surrey Borders Research Ethics Committee (Ref: 19/LO/0778). The studies were conducted in accordance with the local legislation and institutional requirements. The participants provided their written informed consent to participate in this study.

## Author contributions

NS: Formal analysis, Methodology, Writing – original draft, Writing – review and editing. SO’K: Investigation, Methodology, Project administration, Supervision, Writing – review and editing. SH: Formal analysis, Writing – review and editing. MS: Conceptualization, Investigation, Writing – review and editing. RM: Conceptualization, Funding acquisition, Project administration, Supervision, Validation, Writing – review and editing.
