# Supplementary material for: Re-imagining crisis care: experiences of delivering and receiving the Assured brief psychological intervention for people presenting to Emergency Departments with self-harm
Source: Front Psychiatry. 2024 Mar 26;15:1271674. doi: 10.3389/fpsyt.2024.1271674 (PMC11004764; doi:10.3389/fpsyt.2024.1271674)
Supplement: Supplementary file 1 [file DataSheet_1.docx]

**Interview schedule: Patient interview**

Prep beforehand: familiarise yourself with the participant’s recordings/ safety plan/ meeting records etc.

| - Introduce self - Aim of the interview: - Confidentiality, check participant is still happy to participate in the research - Stress the following points: - We are interested in what is important to you - There are no right or wrong answers, it is your perspective that we are interested in. - You are not obliged to answer; if you don't want to talk about a subject, please say so and will move on   General guidance:   - Prompts to check participant is happy to explore further sensitive issues: *Is it alright if we talk about that?* - Mirror wording used by the person - Useful prompts:   • Can you give me an example of…  • Could you describe X further?  • What was the most helpful thing about X?  • What was it that gave you that feeling? |
| --- |

I would like to ask you about your visit to the ED on (date), how the meeting with the mental health practitioner went and about any contacts you have had with the ED team since.

**ED ASSESSMENT**

- Could you tell me about your meeting with the mental health practitioner in the A&E on (date)?
- What worked well for you? What didn’t work so well for you? *(Ask for specific examples)*

***Narrative interview***

- Did you feel you were able to speak openly about what happened to bring you to A&E?

***Question design/communication***

- How relevant were the questions that the practitioner asked you?
- When [practitioner] was asking questions, was there anything in particular you noticed about the way he asked you questions? The way he/she phrased things?

***Safety plan***

- Did you talk about a safety plan? If so, can you tell me about it?
- Were there parts of the safety plan that were helpful?
- Were there parts of the safety plan that were unhelpful?
- Have you used the safety plan since leaving the ED? How?
- Have you received a safety plan from the ED before? If so, how does it compare?

***Evaluating ED assessment***

- What was the most important outcome of the meeting for you?
- Was there anything they didn’t suggest that may have been helpful for you?
- If relevant: How did your experience with this practitioner compare with previous experiences in the ED?

**FOLLOW-UP CONTACTS**

- Have you had any contact or follow-up meetings with the same practitioner you met in the ED?

***IF NO:***

- Explore – what happened? Anything that could have made it possible for them to attend?

***IF YES:***

- Can you tell me about those meetings/calls?
- Did you discuss your best hopes? How did you feel about what was discussed?
- What was helpful/not helpful?
- Did you review the safety plan?
- Did you discuss resources outside of the NHS?
- What did you think about the timing of the follow up sessions? Were they too soon? Too late?
- Did you miss any meetings? Could you tell me about that?
- How did you feel about the follow-up meetings coming to an end?
- Did you discuss your care after the end of the follow-up meetings? *Explore handover of care*.

What was the most important outcome of the follow up sessions for you?

***Delivery of sessions***

- How did your sessions take place (f2f, phone, video)? Would you have preferred a different approach?

**RELATIONSHIP**

- What was your impression of the practitioner in your first meeting?
- How do you feel about your relationship with the practitioner?

How did you feel about meeting with the same practitioner that you met in the ED?

**INVOLVEMENT OF OTHERS**

- Was a trusted other involved in any meetings with you?

***IF YES:***

- Who? How were they involved? How did you feel having them involved?

***IF NO:***

- Would you find it helpful to have someone involved in the meeting with you? How?

**LETTERS**

- Did you receive any letters from the practitioner? If yes: can you tell me what you thought about them?

**CRISES/ SUPPORT SINCE LEAVING ED**

- Have you experienced any periods of distress/crisis since participating in the study? How have you managed them?
- Have you been back to the ED? *If so, explore.*
- Have you seen a GP or other mental health practitioner about self-harm? What happened?

**EXPERIENCE OF TAKING PART IN RESEARCH**

- How did you find taking part in the research? Explore completing measures, sessions being recorded, receiving postcard
  - What do you think of the questions we ask you during the research assessment/ our conversation about your mental health.
  - Do you think the questions capture changes in your mental health?
  - What do you think has changed in your life as a result of taking part in the study?
