# Supplementary material for: Re-imagining crisis care: experiences of delivering and receiving the Assured brief psychological intervention for people presenting to Emergency Departments with self-harm
Source: Front Psychiatry. 2024 Mar 26;15:1271674. doi: 10.3389/fpsyt.2024.1271674 (PMC11004764; doi:10.3389/fpsyt.2024.1271674)
Supplement: Supplementary file 2 [file DataSheet_2.docx]

**Interview schedule: Practitioner interview**

| - Introduce self - Aim of the interview: - Confidentiality, check participant is still happy to participate in the research - Stress the following points: - We are interested in what is important to you - There are no right or wrong answers, it is your perspective that we are interested in - You are not obliged to answer; if you don't want to talk about a subject, please say so and will move on   Prompts to check participant is happy to explore further sensitive issues: *Is it alright if we talk about that?* |
| --- |

I would like to ask you about your experience of delivering the ASSuRED intervention to people recruited into the study.

**Feasibility of intervention**

1. Could you tell me about how you have found delivering the intervention with people who have presented with self-harm?

- What worked well?
- What didn’t work so well?

Make sure every component of the intervention is covered:

- Narrative interview – how did it compare to the usual assessment?
- Safety plan
- 72-hour phone call
- Solution focused follow-ups
- Handover of the safety plan
- Letters

1. Did you include a trusted other in any of the sessions? Explore
2. How did you organise the logistics of the follow ups? (E.g. scheduling, format – intervention interval input from researchers) Challenges / ways to support
3. Were the sessions face-to-face or remote? Explore
4. How did you feel being recorded?

**Delivering intervention within NHS context**

1. How was it different with different patients? And different presenting problems? *E.g. CAMHS/adult/complex cases/people who didn’t engage*
2. How did you find delivering the intervention in the context of the NHS system and other services?

Prompts: Did you have participants who were linked with secondary services? How did that work? What were the challenges? How did you overcome them?

Explore: L*inks with GP’s, communication with other teams e.g. crisis teams/secondary services/voluntary*)

1. Were there challenges in delivering the intervention within the team/organisation?
2. What has supported your delivery of the intervention (personally, within the team, in the organisation)
3. What aspects of the intervention would you change? In what way?

**Training and supervision**

1. How did you find the training? Did you feel ready to deliver the intervention after the training?
2. How did you find the manual?
3. How have you found supervision? Explore: content, frequency, preferences

**Influence of practice**

1. Are there ways in which your practice has changed in working with people presenting with self-harm since completing the training?
2. What would you continue doing in your practice from this new approach?
3. Is there anything else that we haven’t covered that you would like to share about your experience of taking part in the ASsuRED study?
